# Hustling and suffering: Male sex workers and HIV interventions in Kenya

**DOI:** 10.1080/17441692.2025.2501167

**Published:** 2025-05-13

**Authors:** Emmy Kageha Igonya, Eileen Moyer

**Affiliations:** a African Population Health and Research Center, Nairobi, Kenya; b Department of Anthropology, University of Amsterdam, Amsterdam, The Netherlands

**Keywords:** Social suffering, HIV activism, sex work, hustling, Kenya, SDG 3: Good health and well-being, SDG 5: Gender Equality, SDG 8: Decent work and economic growth, SDG 10 Reduced inequalities

## Abstract

Participating in and working for HIV interventions is both a source of both pride and suffering for many men who have sex with men (MSM) who engage in low-paying sex work in Kenya. Drawing on ongoing intermittent ethnographic research conducted among MSM sex workers since 2010, we analyse the relationship between hope and resilience on one hand, and narratives of suffering and hustling on the other. We show how HIV technologies that provide spaces for visibility and mobilising, such as new treatment regimes, accompanying support groups and training programmes, as well as activist led organisations, allow MSM sex workers to contribute to national and global HIV responses with a sense of both pride and shared suffering. We argue that pride, suffering and hustling are central to male sex workers’ identity, solidarity and resilience. Attempts to build resilience among MSM sex workers and other highly marginalised people at continued risk for HIV would be advised to take their complex ambivalences towards health and rights-based interventions into account.

On a June 2016 visit to Freedom Corner, a run-down bar located in Nairobi's central business district, we meet up with three male sex workers. Our companions identified as men who have sex with men (MSM) or *kuchus*, a term borrowed from neighbouring Uganda and considered safer for public use. It had been two years since our last visit with the Freedom Corner Support Group, formed in 2010 by HIV-positive MSM sex workers who had recently begun treatment (Igonya & Moyer, 2016).

The support group provides social financial and emotional support, and nursing care, while advocating for income earning opportunities and medical treatment. Although we had maintained contact through phone calls and online chats, we decided an in-person visit was needed to assess how they had fared amid Kenya's increasing HIV interventions targeting MSM. After catching up, we asked how their lives had progressed over the previous three years. They reported that they continued to ‘suffer’ despite what they characterised as a significant influx of resources for MSM in Kenya over the preceding decade. While most had access to antiretroviral drugs and better health care in general, their material conditions remained dire. They continued to face emotional and physical abuse, were still targeted by police, and had insecure housing.

In 2010, we noted how stories of suffering shaped MSM identities, as they commonly invoked ‘sufferer’ identities to gain more from interventions or to hustle for material aid (Igonya & Moyer, 2016). Over time, we observed MSM sex workers refining their hustle; some transitioned from merely trading stories for handouts, to securing gigs and full-time jobs in HIV interventions, with others leading their own HIV organisations. However, this increased visibility sometimes hindered their main hustle – sex work. We also noted how new HIV programmes, funding changes and the introduction of preventive HIV treatment (PrEP) affected solidarities within this group. Building on previous work that attempts to understand the effect of HIV interventions over time (Igonya et al., 2022), we seek to untangle this complex interplay of identities, funding and HIV technologies to understand why some MSM sex workers continue to struggle despite years of investment, how they maintain pride in programmes that have failed them, and how their impressive resilience is woven into narratives of suffering and hustling.

NGOs and the Kenyan government’s efforts to include male sex workers and MSM in HIV initiatives is commendable, but many programmes that address economically vulnerable participants result in unintended negative consequences, including the exacerbating of suffering. Although our participants accessed HIV services, participation in MSM-targeted programmes increased their visibility, leading potential clients to see them as HIV-positive and to disengage. They lamented that, even though they had an enhanced knowledge of HIV treatment and prevention, their clients, many of whom were secretive about their sexual preferences, were unaware of the prophylactic benefit of antiretroviral drugs.

Since our research began at Freedom Corner, the space and the group have been tied to a community-based organisation, which we refer to as Key Pops United or KPU. Founded by male sex workers and led by and for MSM, KPU has used donor funds to provide extensive health services to marginalised MSM, advocate for by MSM sex workers’ rights in Kenya, and transition from an organisation of service users to one of service providers, including operating a clinic for HIV and STI treatment. While KPU has raised awareness about MSM sex workers’ economic and psychological issues, its greatest success has been expanding access to antiretroviral treatment and PrEP for sex workers.

Despite KPU’s successes *kuchus* at Freedom Corner explained that their association with KPU had resulted in a loss of clientele and reduced income. Nancy^1^ (he/him/his) recounted how a client, upon learning he was KPU member, presumed Nancy was HIV-positive. Carol (he/him/his) echoed this sentiment: ‘Potential clients do not want HIV-positive sex workers; because of KPU, clients think that all MSM sex workers in Nairobi are HIV-positive’. As Thomann et al. (2022) and colleagues have demonstrated in their research on HIV self-testing practices among male sex workers, those who appear and can prove they are health, i.e. HIV negative, often earn more money.

## Suffering and hustling

Nancy described the condition of MSM sex workers like him using the Swahili-English slang term ‘*masufferer’,* an identity commonly claimed by members of Nairobi's underclass. More than simply translating as ‘sufferers’, it refers to the togetherness of people bound by shared experiences of economic deprivation, stigmatisation, and marginalisation (Ojwang, 2017). The term invites a reflection on how material poverty is intertwined with injustice, poor physical health, and social, moral and spiritual malaise. As Ojwang (2017) and others have shown, the category *masufferer* offers theoretical insight ‘from below’, allowing for a more comprehensive understanding of the sociality of suffering as something that is collective and shared. Among the Freedom Corner *kuchus*, the term's moral force helped foster relationships among people who shared similar hardships, going beyond the biosocial categories utilised by HIV support groups focused on biomedical intervention to recognise and foster bonds among those living with economic deprivation and multiple forms of stigma.

While it is difficult to escape such systemic injustices, dreams of escape are not wholly stymied. For many, the *masufferer* identity also invites what Thieme (2021) and others have called ‘hustle’. Hustle is a devised survival strategy that may involve various activities or anything people do to get money (Thieme, 2018; 2021; Monteith & Mirembe, 2021). Risk, including the risk of selling sexual favours, is often inherent to hustling. Researchers working in Kenya have documented the ways that hustling is constructed and deployed in various vocabularies and practices (Mwaura, 2017; Thieme, 2018; Thieme et al., 2021; van Stapele, 2021). While much research focuses on hustling *activities*, here we want to foreground hustling’s relationship to the *masufferer* identity among male sex workers who see both their sex work and the work they do for HIV interventions as forms of hustling that together constitute the agentive side of their *masufferer* identity. Similarly, van Stapele (2021) shows how collectives around gang identity come into being in relation to forms of illegal hustling practices that allows men to provide for their families and proclaim their masculinity. For Freedom Corner MSM, the ability to mobilise as *masufferer* is critical to their success as hustlers in both contexts. Governmental and non-governmental organisations in Kenya are frequently willing to provide favours to groups of young people (Ojwang, 2017), including male sex workers, who can work on their behalf, raising awareness and fostering community support.

We use an intersectionality framework (Collins, 2015; Crenshaw, 1989) to highlight that it is a combination of identities – MSM, sex worker, HIV positive and negative status – which contributes to participants’ suffering, hustling and resilience. Though they have differences based on and HIV status, often, they share a lack of access to basic services and opportunities including education, welfare, health and sources of income (Armisen, 2016; Epprecht et al., 2018; Muzenda & Kessman, 2017). They also share experiences of stigma and discrimination, violence and criminalisation. These experiences have shaped their survival strategies and relationships (Okal et al., 2009).

Owing to a high HIV prevalence among MSM, they have been heavily targeted by HIV treatment and prevention interventions (Doshi et al., 2020; Graham et al., 2018; Lorway, 2020; Moyer, 2019). As ‘targets’ of global health interventions, they have been regularly invited to training workshops on behavioural change, treatment literacy, disclosure, and peer education as agents of HIV prevention (Igonya & Moyer, 2016; Igonya, 2017; Shannon et al., 2015). Others have documented the disjuncture between what MSM sex workers have requested and what HIV interventions offer, highlighting the extent to which their wants and needs are ignored (Lorway, 2015; Woensdregt & Nencel, 2020). Lorway, highlighting ‘the growing interdependencies between sex workers and scientific and technical experts’, suggests that the demands and actions of sex workers demonstrate possibilities for resisting and reshaping colonial power relations (2020, p. 402), but our data suggests that such possibilities are limited by the very real structural and financial constraints of sex worker led community action groups, something Lorway also observes (Graham et al., 2018; Kombo et al., 2017). Similarly, Kombo et al. (2023) have illustrated that collaborations between academic research organisations from the global North and community-based organisations in global South that promise to unlock economic opportunities are often limited by the temporality and sparse community focused funding of these collaborations, which tend to prioritise data collection and biomedical treatment (Thomann et al., 2022).

Whereas the humanitarian apparatus often paints MSM sex workers as deserving subjects of aid and intervention due to their medical and economic distress, the *kuchus* at Freedom Corner saw themselves additionally deserving because of the labour they provided to research and intervention programmes targeting them. Sex workers involved in community-based research and mobilisation activities play a crucial role not only in data collection, but also in study design and the recruitment of study subjects (Thomann et al., 2022). Making the distinction between sex workers as aid recipients in desperate need or as providers of labour who shape actively shape the world of research and intervention is critical. This is not only because it describes the realities of global health knowledge production; it reveals how narratives of suffering and hustling can be understood as and mobilised as a demonstration of agency rather than victimhood. It also invites anthropological consideration of how human suffering is understood in local contexts, which may undermine universalist assumptions. Departing from Robbins’s (2013) invitation to abandon the ‘suffering subject’, we call for a situated analysis of suffering and hustling to better understand how agency and resilience may be entwined with the scientific and political projects of biomedicine, the state, and humanitarianism. Against the odds, MSM sex workers in Kenya banded together to form a community-based organisation, navigate the domain of HIV interventions, and help deliver health care to their peers, becoming a model for male sex workers elsewhere in the world. While this should certainly be interpreted as a testament to their resilience, it also demonstrates the ways *masufferer* identities can propel collective action and unexpected forms of hustling. From a theoretical perspective, this demonstrates the agentive power of the invocation of collective experience as an alternative to equating suffering with victimhood.

Drawing on our observations of how *masufferer* identities were articulated and mobilised among male sex workers at Freedom Corner, we highlight the complexities of social suffering and resilience in practice to show how MSM sex workers shape their life stories through mobilisation and other social forms of care. Narratives of suffering play an important role in conversations about the disparities in value systems that create the worlds of both humanitarian relief and street hustlers. Tales of pain and suffering do more than mirror or narrate experience: they are also how MSM sex workers socially place themselves in the world as legal, political, economic, moral, and medical subjects (Kleinman et al., 1997). We examine *kuchus’* articulations of suffering, paying attention to how suffering is entwined with subjectivity in the context of HIV interventions aimed at them and KPU, the organisation they created to relieve their suffering. We also look at how the suffering of MSM sex workers is perpetuated and even exacerbated by the introduction of new HIV technologies like PrEP.

## Study site and methods

The article builds on long-term ethnographic fieldwork in Nairobi, Kenya. Since 2010, the Igonya has conducted research with male sex workers, both HIV positive and HIV negative, while Moyer contributed study design and analysis. Although the article primarily draws from two studies in Nairobi, the second completed in 2018, ongoing engagements with key participants have periodically updated findings relevant to our arguments. The below table provides an overview of the different periods and methods used.

**Table:** 

Year of Data Collection	Site	Participants	Study Design	Methods
**2010–2014**	Nairobi	MSM living with HIV	Ethnography	• 72 Participant observations• 24 IDIs HIV positive MSM sex workers• 8 Life History Interviews• 2 FGDs
**2014–2018**	Nairobi	MSM living with HIV	Ethnography	• WhatsApp check-ins• Participant observation during key community and life events• Participant observation during MSM-related scientific and policy workshops and conferences• Informal Interviews
**2018**	Nairobi	HIV negative MSM sex workers	Qualitative	• 18 IDIs• 4 Observations of PrEP group sessions• In-depth Interviews
**2019-present**	Nairobi	MSM sex workers	Ethnography	• WhatsApp check-ins• Participant observation during key community and life events• Participant observation during MSM-related scientific and policy workshops and conferences• Informal Interviews

From 2010 to 2013, we regularly met MSM sex workers at Freedom Corner, joined them for activist and training activities, and collected data at public hangout spaces. From 2014 to 2019, interactions included intermittent emails, phone calls and occasional face-to-face meetings, including at funerals. Starting in 2013, Igonya increasingly worked with KPU-affiliated MSM sex workers during their research and outreach activities, maintaining contact with key participants via WhatsApp, calls and attending events. Since the 2018 study, contact has continued through similar methods.

Data collection included participant observation, in depth interviews, focus group discussions and informal conversations, both face-to-face and via phone or WhatsApp. Observations took place at Freedom Corner, KPU activities, and during outings with participants to explore their lives beyond sex work. Informal conversations were documented as short notes on mobile phones and expanded into fieldnotes within 24 hours. Additional data came from three FGDs and seven informal discussions with MSM sex workers on PrEP and three in-depth interviews and regular informal discussions with the programme manager of KPU.

The Kenyatta National Hospital/University of Nairobi Ethical Review Committee reviewed the research protocol and provided ethical approval. All participants consented and verbally agreed to participate in the study. To protect livelihoods and ensure security, we use pseudonyms, including for KPU.

## Participation of MSM sex workers in HIV interventions in Kenya

In 2011, the Support for Addiction Prevention and Treatment in Africa (SAPTA) facilitated a training session at which we had our first interaction with MSM sex workers. While the training was designed to promote adherence to HIV treatment among MSM, participants requested an extra session to discuss psychosocial concerns affecting treatment adherence (Igonya & Moyer, 2016). When asked to explain what they meant by the facilitator, they mentioned homelessness, rejection, isolation, stigma, a lack of economic possibilities, societal violence, HIV-positive status, and sex worker status. Despite agreeing that these were highly important issues that limited people’s ability to take medications as directed, the SAPTA facilitator denied the request. One participant, Maureen (he/him/his), discussed her disappointment:

MSM sex workers face a triple stigma: HIV-positive status, sex work, and MSM. Sex workers are struggling to make ends meet … However, there is insufficient funding for HIV interventions aimed at HIV-positive MSM sex workers. The organizations that get the funds are unconcerned about our plight. They tell us to take HIV drugs and use condoms, but we are hungry. Except for transportation allowances granted during training, organizations do not hire sex workers.

Maureen's comments are typical of the critiques levelled by MSM sex workers. Well-funded programmes primarily focus on providing HIV treatment and related services, such as testing for HIV and STIs, HIV counselling and behavioural modification. Participants receive transportation allowances to encourage their involvement. While participants in the study understood the biomedical value of such interventions, they critiqued the limited attention given to income-generating opportunities and material support. Even when presented in humanitarian and rights-based terms, biomedical treatments mostly centred technological solutions to narrowly defined health concerns while neglecting structural factors that contribute to disease and poor adherence. This is the first time we observed the invocation of a *masufferer* identity in an intervention setting. Maureen and the others present understood that their presence at the training was valuable to SAPTA and they wanted something in return: recognition that their subjectivities exceeded the medicalised slot they were invited into.

MSM sex workers were often disturbed by the biomedical emphasis of therapies, which they believed ignored the source of their misery. This caused conflict between HIV interventionists and HIV-positive MSM sex workers. The disappointment also resulted in and cemented new social relationships, including the formation of the Freedom Corner support group. Importantly, the support group grew into an efficient HIV response network as it expanded, allowing them to cultivate critical (income-generating) partnerships with key research and interventionist organisations that recognised and compensated them for their labour (Igonya, 2017; Igonya & Moyer, 2016). Freedom Corner served as an important site for the reflecting on the relationship between narratives of suffering, labour and *masufferer* identities.

Even though the group was formed to fill gaps left by existing MSM interventions and programmes, their ability to mobilise sex workers to seek HIV testing and treatment resulted in them being highly sought after by key government agencies such as the National AIDS Control Council (NACC), the National AIDS and STI Control Programme (NASCOP), as well as national and international civil society organisations. Over time, the MSM sex workers of Freedom Corner grew to perceive themselves as useful to the humanitarian apparatus that was targeting them. They understood this also in biological terms: their HIV status and exposure to HIV risk increased the value of their blood and semen for biomedical study. They knew that they ‘checked’ three boxes highly valued by HIV interventions targeting ‘most at risk’ populations: sex worker, MSM and HIV-positive. This intersectional biosociality allowed them to benefit from the growing need for MSM participation in HIV programmes. While they routinely offered themselves as participants, they learned that it was more profitable to mobilise others to participate, or to work as peer mentors for organisations that executed MSM programmes. With increased focus on priority populations, the *kuchus* of Freedom Corner embraced any chance to assist with interventions, seeing this as a step towards employment.

December 4, 2011. Carol (he/him/his) and a friend arrived at Freedom Conner around 7 pm Throwing an envelope on the table, Carol took a seat and remarked, ‘I have more than ten certificates, but they are of no help to me. The certificates aren't getting me anywhere’. In the envelope was a certificate for completing a training session on HIV/AIDS treatment adherence and prevention, organised by a biomedical research programme. Carol was one of the most educated members of the Freedom Corner group, and he was one of the few who attended training sessions offered by several *kuchu*-targeting organisations. Participants in training typically received daily transportation incentives, ranging from KES 200 to KES 500 (2–5 USD) and a certificate. Carol initially participated in training to better his work prospects but became increasingly frustrated. Many, like Carol, voiced dissatisfaction with the organisations that had targeted them, complaining that they were used as ‘window dressers’ to garner donor funds. Josephine (he/him/his) explained, with exasperation:

You see, these organizations get money from donors because of us, but we do not benefit. What we really need is opportunities to enable us to buy food and pay for housing. Most of us are homeless. Freedom Corner and two parks are our home. During the day, most of us sleep or live at the parks from morning to five in the evening and then move to Freedom Corner. And that is the life of the majority of us. We do not underestimate these organizations. However, they should also pay attention to our life struggles. A hungry and homeless person cannot take medication and cannot tell clients to use condoms because you are not sure if the client will accept HIV-positive sex workers. Clients have abandoned many of us. We know projects provide jobs to educated people, but they have other jobs like cleaning and security that we can do.

Here we see Josephine referring to the loss of clients that has come about due to him visibly engaging with and supporting HIV intervention activities. He is explicitly not asking for a handout, but rather a job like cleaning or security that someone less educated might be able to do. More than a plea for work, Josephine highlights what is understood as a moral obligation to provide alternative income generating opportunities for those who publicly share their stories of suffering to allow HIV organisations to operate.

MSM sex workers who were on PrEP interventions made similar complaints. The introduction of PrEP brought HIV-negative MSM sex workers who had been on the periphery of HIV interventions to the centre of HIV interventions. PrEP was introduced to reduce chances of HIV infection (Bavinton et al., 2018; Liu et al., 2016). Karen (he/him/his), a HIV-negative sex worker on PrEP who was mobilising others like him to form a support group, saw himself as an ally of HIV-positive sex workers. While appreciating having access to PrEP and KPU’s support groups, Karen noted that most organisations were concerned with promoting adherence, ignoring the wider concerns of many HIV-negative MSM sex workers (Lorway, 2015; Moyer, 2019; Moyer & Igonya, 2018). Although they were aware of the dangers of not following pharmaceutical regimens and outspoken about the importance of adherence, they were also empathetic towards people who failed to adhere.

## Nothing about us without us

The Freedom Corner sex workers were aware of their collective value to national and global HIV response initiatives and demonstrated this by participating in HIV interventions as a collective. Indeed, part of their hustle was working collectively. Showing up together at events helped ensure their safety, but they were also aware of the theatrical effect they made as a group of effeminate men moving collectively in public space. Their strong sense of community has made it relatively straightforward for NGOs and other interested parties to collaborate with them. A phone call from a peer mobiliser could quickly result in a meeting attended by most of the members, collective participation in public rallies or NGO-sponsored training sessions on short notice.

They were generally aware of the dangers of not following pharmaceutical regimens. They were outspoken about the importance of adherence while staying empathetic towards people who failed to adhere due to financial or psychological issues. In so doing, Freedom Corner emerged as a place where people could forge solidarity based on their HIV status, sexuality, sex work, shared sense of suffering, and views about hustling. They also began to collectively position themselves as crucial players in HIV interventions. This new dynamic, in which targets of global health initiatives became participants in the intervention itself, was fostered by several variables, including the mantra that has guided HIV activism since the emergence of the AIDS epidemic: ‘Nothing about us without us’.

As MSM sex workers began to organise collectively, their political consciousness and sense of solidarity expanded, as did their understanding of activism around HIV and sex work on a local and global scale. Many interventions included a research component, so some MSM and female sex workers from Freedom Corner were also trained as fieldworkers. The first study they were involved in focused on male and female sex workers in four African countries; this culminated in 2011, in a session to disseminate the findings, where they emphasised the importance of their contributions to worldwide research. After the presentations of two sex workers, applause was replaced with chants of ‘nothing about us without us’. During the break, when we congratulated the speakers on their presentations, one replied, ‘I'll be a consultant in this field in three years. I don't have the academic credentials, but I am an HIV-positive sex worker acquiring experience’. This remark perfectly describes the atmosphere of the period.

The HIV infrastructure in Kenya, which in 2009 began targeting so-called critical demographics, such as MSM and sex workers, presented a unique structure for hustling and a sense of hope, as MSM sex workers positioned themselves as representatives of their kind. They could meet the ‘nothing about us without us’ requirement of many HIV programmes, also articulated in two principles: ‘greater involvement of people living with HIV/AIDS’ (GIPA) and ‘meaningful involvement of people living with HIV/AIDS’ (MIPA). And they were essential in recruiting MSM sex workers for research and interventions, as in speaking for the efforts at national and international forums. Their hustle contributed value to HIV research and treatments aimed at MSM and sex workers, and they drew attention to that value through activist scripts of solidarity, inclusiveness and economic justice.

Establishing KPU was an exceptional result of the Freedom Corner support group's collective work. KPU was Kenya's first officially registered community-based organisation founded and headed by HIV-positive MSM sex workers. Despite the prohibition of homosexuality at the time (2019–2019), MSM and female sex worker-led organisations proliferated, mostly in Kenya's major cities of Mombasa, Kisumu and Nairobi. KPU embodied the activist ideal: male sex workers would design and implement intervention programmes specifically for themselves and their community. Initially, KPU concentrated on mobilising MSM and encouraging access to HIV testing, treatment and psychosocial support, and this focus allowed them to obtain donor financing for HIV-related activities. Their presence in the media and at (inter)national conferences increased the collective value of MSM sex workers, raising awareness among both donors and sex workers of MSM sex workers’ importance to the success of HIV initiatives.

Following their initial success, KPU was able to raise further funds, allowing them to focus on rights-based campaigning, such as demanding legal rights for MSM and sex workers, and working with female sex workers to decriminalise sex work as a whole. In 2015, KPU provided clinical services and coordinated research operations, in addition to providing psychological support and engaging in advocacy activities. MSM sex workers affiliated with KPU also attended international HIV conferences, where they contributed to both activist and scientific debates, sharing the stage with high-ranking scientists and policymakers from Kenya and the rest of the world while raising funds for their cause.

As KPU’s popularity and success grew, so did the expectations and goals of the Freedom Corner support group. However, only a handful realised the promise of earning more money, as they were given long-term job contracts. Some worked occasionally as peer educators and daily allowances were granted to those who participated in lobbying and mobilisation efforts. KPU and the University of Manitoba collaborated to hire several Freedom Corner support group members, who acquired considerable training in social science data collection methodologies, increasing their value as collaborators with the university and other research institutions. These were not inconsequential developments, but the job opportunities entailed by KPU’s activities were insufficient to eliminate the economic obstacles that most *kuchus* faced. Significant tensions developed between those who appeared to have profited the most and those who had received no job or had only been hired as peer educators and paid a low monthly allowance of 3500 KES (35 USD), as mandated by NASCOP (NASCOP, 2014).

The existence of KPU brought to light previously ignored disparities among sex workers, including that some members had greater social capital related to educational level, English proficiency, and class background. While the Freedom Corner group's collective identity had been centred on physical, psychological, and economic hardship, those members of the group were better positioned to successfully hustle in this new economy. The others, left behind at Freedom Corner, frequently lamented their inability to establish their own CBOs due to their lack of education, and they expected their more educated compatriots to stand with them. Ultimately, KPU, despite being sex worker led, was unable to affect the structural adjustments necessary to ease the suffering of the less fortunate from Freedom Corner. This is hardly a critique, as KPU relied on the same funding sources as other organisations working with MSM and sex workers, were subjected to the same reporting and evaluation mechanisms, and expected to produce evidence suitable for international analysis (Biruk, 2012; Lorway et al., 2019). This meant that despite desires to assist poorly educated MSM sex workers, KPU ended up hiring primarily those who could communicate in English and master data collection skills. Further, KPU was.

## The socio-economic risks of visibility

MSM sex workers have consistently reported that their HIV-positive status worsened their suffering by jeopardising their livelihoods. Clients often abandoned workers suspected of being HIV positive status, despite advances in treatment and evidence that people on antiretroviral therapy with undetectable viral loads cannot transmit HIV. Many clients lacked knowledge about HIV prevention, including the protective benefits of PrEP, and assumed all KPU-linked sex workers were HIV positive. Condom use, advised to prevent re-infection and STIs, often led clients to suspect HIV-positive statis, creating ethical dilemmas for sex workers reliant on this income.

Clients were seen as potential long-term arrangements, making it necessary for sex workers to conceal their HIV status and avoid suggesting condoms use. client. Stella (he/him/his) explained, ‘What else can you do? You're hungry, need food, don't have a place to sleep, and don't have any money’. Sofia (he/him/his) added, ‘Disclosing [your] HIV-positive status or initiating condom use is *kumwaga unga* [literally: wasting flour, a colloquialism for passing up an opportunity]’. During 2019 focus group discussions, most participants recalled losing clients after suggesting condom use.

At the 2015 International Day to End Violence Against Sex Workers, Jedida (he/him/his), a Freedom Corner co-founder shared how contracting an STI had cost him more to treat than he had earned from the client who infected him. He explained,

The moment you mention condom use, clients start asking whether you are HIV-positive. I lost many clients to friends who are HIV-positive but told clients they were HIV-negative or never mentioned using condoms. Since then, I don't disclose my HIV status and never ask clients to use condoms. Jobs are scarce. Now, if you get clients for sex work, you don't want to lose them.

Clients were few, and sex workers struggled to make ends meet, but sex work remained the primary hustle or way to earn a living as other work was often inaccessible. Jedida, for instance, lost his restaurant job when his sexual orientation was revealed, forcing him to return to sex work. Before KPU’s founding, but KPU’s visibility this. By 2014, KPU's association with HIV positivity had grown in Kenya and globally. Nancy (he/his/him), an educated sex worker from Freedom Corner, secured a position with KPU and supplemented his income with sex work. However, he lost a promising online client who, upon learning of Nancy’s KPU affiliation assumed he was HIV positive. Nancy reflected, ‘I thought clients from abroad are informed. No! All clients are the same, whether from Kenya or elsewhere. Why did he leave me when we could have used condoms? […] You don't have to disclose to the client … But there is still a problem when it comes to requesting condom use.’ Narratives like Nancy’s are common and highlight the stigma MSM sex workers face due to KPU’s association with HIV positivity. Clients were sceptical about the effectiveness of HIV antiretroviral treatments and assumed that anyone connected to KPU was HIV-positive, pressuring even KPU-linked HIV-negative sex workers taking PrEP to forgo condom use.

## PrEP: Escaping or generating social suffering?

PrEP has been available in Kenya since 2016. HIV interventions seek to identify persons who are at a higher risk of contracting HIV and encourage them to take antiretroviral medications to reduce their chances of infection. PrEP programmes have primarily targeted MSM and female sex workers. Given that the efficacy of such interventions has been dependent on regular pill intake, PrEP clubs have emerged to encourage adherence. Prior to PrEP, HIV-negative MSM sex workers were generally excluded from HIV interventions and peer education training. ‘We are always left out of HIV activities’, Karen explained, ‘and there was some coldness towards us [HIV-negative MSM sex workers] from those who were HIV-positive’.

The introduction of PrEP significantly altered the intervention landscape. As being HIV-negative came to be valued in interventions, new biosocial ties were formed and others disturbed. When we first met, Karen, an MSM peer educator who is on PrEP and who works for an NGO that implements PrEP interventions, he announced that he was establishing PrEP support groups. First, Karen was concerned about the negative effects of being associated with KPU, including the possibility of lost economic opportunities for MSM sex workers and the way HIV-negative MSM sex workers were positioned in HIV interventions. He wanted to keep a separation between HIV-positive and HIV-negative MSM sex workers.

As KPU gained prominence in Kenya, it was better positioned to mobilise MSM sex workers, including for health and human rights initiatives, regardless of HIV status. For example, KPU has played a key role in mobilising HIV-negative MSM sex workers for PrEP interventions and studies. Furthermore, because KPU had previous expertise with psychological support and peer education, they were able establish and lead PrEP clubs. While the clubs were intended to support the success of PrEP programmes, they also provided opportunities for HIV-negative *kuchus* to form and benefit from new biosocial ties that fostered a sense of belonging, while connecting with larger HIV-positive MSM sex workers.

However, according to Karen the disparities between the two groups made it hard for HIV-negative people to benefit from clubs organised by HIV-positive organisations. He explained:

MSM sex workers have issues affecting them … basic needs like housing, food, and other problems can affect adherence to PrEP. We need to support those who do not have a place to stay or lack food. I think it is time donors pay attention to these issues. KPU and the Freedom Corner group have dented the MSM sex workers’ identity because of their HIV-positive status. All MSM sex workers are considered HIV-positive because of KPU. This is not suitable for sex work. A majority of us have no other job – sex work is our only source of income. With PrEP, we have a chance to redeem our image and get clients if we do not associate with KPU.

Here we see that HIV-negative MSM sex workers have similar concerns as HIV-positive sex workers. Karen suggests that those with a HIV-negative positionality would prefer to focus on material and economic well-being rather than HIV status, a sentiment shared by HIV-positive sex workers. Karen also envisioned a group formation that would dissociate MSM sex workers from an HIV-positive status hoping this would secure more clients for those who were negative and on PrEP. Despite this desire, he also reported that, ‘clients took off when they saw one with PrEP. ‘You know PrEP is ARVs. They [clients] think those with PrEP are HIV positive’.

## Conclusion

This article attempts to make sense of ongoing struggles among MSM sex workers in Nairobi despite years of investment by HIV programmes. Focusing on narratives of suffering and hustling that are intertwined, we have demonstrated their resilience and the pride they take in their involvement in the development of these programmes despite their failure to address underlying causes of suffering. There is general recognition that embracing the *masufferer* identity can be advantageous if one can tell a convincing story of suffering to the right people. Over time, the *kuchus* of Freedom Corner have come to identify as *masufferer* deserving of both medical and socioeconomic intervention. This is not only because they are poor, but also because they see themselves as hustlers whose sexual, biological, political and confessional labour undergirds the ongoing success of the MSM-intervention apparatus. In the context of our research, they made it clear to us and those responsible for the various interventions targeting them that they understood the value of their stories and organising labour: without their disease risk, ability to organise and willingness to turn out for interventions and protests, HIV programme funds would cease to flow. Perhaps the most critical work they undertake for the aid sector is openly discussing their experiences in ways desired by the industry. Their confessional experiences evoke sympathy and help sustain the humanitarian apparatus that was designed to save their lives (Nguyen, 2010). Similarly, their experiences, often captured in sound bites, images and film clips, are used as qualitative ‘evidence’ by organisations wanting to demonstrate that their initiatives’ effectiveness. The humanitarian apparatus provides the context for new forms of hustle that accompany but rarely replace the regular hustle of sex work. Like their *masufferer* identity, these hustles are key to MSM sex workers sense of pride and resilience.

We have sought to shed light on the limitations of HIV interventions that target MSM sex workers in Kenya, and on the emergence of new solidarities among those targeted by HIV programming (Moyer, 2019; Moyer & Igonya, 2018). Despite intensive investments in HIV treatment and prevention infrastructures designed to reach MSM and other key populations, including sex workers and injecting drug users, marginalised members of these groups continue to ‘suffer’ while also forging new solidarities that afford resilience. HIV medications allow them to survive, but hardly to thrive. While a few male sex workers have managed to find work through affiliating with community-based organisations, NGOs, and clinics targeting key populations, those with less education, have reaped little economic reward for their labour. Our research shows that as their association with HIV-prevention activities increased, their income suffered because clients were less likely to seek their services. Their visibility hampered the selective hiding of identities, forcing them to contend with clients’ presumptions about their HIV status.

An intersectional analysis highlights how sexual orientation, sex work and HIV status combine to exacerbate stigma, discrimination and suffering, but also that neither antiretroviral treatment nor PrEP uptake can eliminate stigma nor reassure clients seeking HIV-negative partners. These biosocial intersections result in certain key populations being valued in particular ways by HIV interventionists who are under pressure from funders to reach high numbers of people at risk. Sex workers, especially more marginalised sex workers, whether male, female, or trans are often easier to reach through HIV programming than others might be because their social capital does not allow them the luxury of privacy and they are often willing to do the low-paid and precarious community mobilisation work that many HIV interventions require to succeed (Moen et al., 2012). Those with greater social capital are often the first to benefit from employment opportunities even within sex worker led organisations. While we do not want to underestimate the resilience of the MSM sex workers whose lives we have followed for over a decade, we are also hesitant to celebrate it, knowing, as we do, that this resilience is largely born out of socialites that have emerged in the face of extreme exclusion and prejudice.

We have also observed that HIV status and use of HIV medications may contribute to a breakdown of solidarity among some MSM sex workers, with some of those using PrEP medications insisting on not being lumped together with those using antiretroviral medications for treatment. Yet both HIV-positive and HIV-negative MSM sex workers expressed similar concerns: economic insecurity, psychological distress in the form of grief and mental anguish, and a need for more from HIV interventions than what was provided. Perhaps it is not reasonable to expect HIV interventions to answer all the needs of MSM sex workers or those of the other key populations they target. Yet, for many who fall into these public health categories, free HIV clinics are one of the few places where health care services are available.

While the inclusion of MSM sex workers in HIV interventions may not have addressed their economic, social and psychological suffering adequately, the forging of solidarities around *masufferer* identities created feelings of pride and meaning in their lives. As sufferers and hustlers, MSM sex workers have not been passive beneficiaries of HIV interventions. They have planned, pursued and provided HIV services. Taking up the call of ‘nothing about us without us’, they refused the label of victim and embraced their capacity to address their own suffering through actions of solidarity and support, including the establishment of KPU. KPU filled a need that other HIV initiatives targeting MSM sex workers could not (Lorway, 2020), even if its financial resources have been limited and they have been unable to meet all the needs of all their members, most notably those with low levels of education like the Freedom Corner *kuchus*. Addressing the needs of MSM sex workers, including health, social, economic and security concerns, is a critical component of the work that KPU has done. The key role they play in the community has become even more apparent over the last five years as queer people in Kenya have faced increasing backlash because of legal decisions and changing political regimes. At the same time, international funding for HIV interventions has steadily declined because of shifts in global priorities, not least of which was COVID-19 (Kimani et al., 2020; Odinga et al., 2020).

After discovering their ability to contribute to and manage HIV interventions, MSM sex workers claimed considerable and expanded involvement in HIV interventions. Despite the limitations of HIV programming in general and of KPU in particular, the Freedom Corner *kuchus* embraced their *masufferer* identities to reimagine their biopolitical positionality and relevance in local, national and global HIV responses. They took a strategic decision to collaborate as *masufferer*, regardless of whether they were HIV-positive or negative. As intervention targets, they chose to enter an alliance with HIV interventionists by both participating in and contributing to the success of their programmes. As MSM sex workers they capitalised on the value of their HIV-positive status, which gave them a sense of power, agency and resilience.

## Note

1 Most study participants (gay and transgender) use female names while at Freedom Corner. Therefore, in accordance with their preference, we have adopted female pseudonyms to match participants' chosen sex work names.
